# Seasonality in Polyps of a Tropical Cubozoan: *A latina* nr *mordens*

**DOI:** 10.1371/journal.pone.0069369

**Published:** 2013-07-26

**Authors:** Robert Courtney, Jamie Seymour

**Affiliations:** 1 School of Marine and Tropical Biology, Faculty of Science and Engineering, James Cook University, Cairns, Queensland, Australia; 2 School of Public Health, Tropical Medicine and Rehabilitation Sciences, Faculty of Medicine, Health & Molecular Sciences, James Cook University, Cairns, Queensland, Australia; 3 James Cook University, Cairns, Queensland, Australia; University of Hamburg, Germany

## Abstract

*A latina* nr *mordens* have been located in large predictable spawning aggregations near Osprey Reef in the Coral Sea eight to ten days after a full moon; however, polyps have never been located *in-situ*. The polyp stage contributes to the abundance of medusae through asexual reproduction and metamorphosis, and may influence the periodicity of medusae by metamorphosis of the polyp. To elucidate the relationship between medusae periodicity and polyp ecology, polyps were exposed to thermal and osmotic treatments in order to determine the theoretical environmental limits to their distribution. Maximum fecundity occurred in thermal treatments of 21 to 25ºC and the theoretical minimum thermal requirement for population stability was approximately 17ºC. Polyps were also exposed to five feeding regimes and fecundity was found to be positively correlated with feeding frequency. Thermal and osmotic variations did not induce metamorphosis in this species, however, reduced food did. The implications of asexual reproduction and cues for metamorphosis in relation to population dynamics of this species are discussed.

## Introduction

Tropical Australian cubozoa exhibit marked seasonality predictably arriving in large numbers in the coastal waters of North Queensland annually [1]. During this period the medusae grow to sexual maturity, spawn, and subsequently disappear (presumably dying) [1,2]. The majority of cubozoa research has focused on coastal medusae primarily due to their painful and potentially fatal stings to humans [2–5]. Cubozoa polyps, which do not have a direct impact on people, have been far less explored.

Currently, what is known about cubozoa is that they have a metagenic life cycle and undergo an alternation of generations between an asexually reproducing, sessile, benthic, polyp and a sexually reproducing, motile, medusa [1,2,6–11]. The majority of research on cubozoa has been conducted on the medusa stage because, for most species, the location of the polyp stage is unknown. Cubopolyps have only been found *in-situ* for two species: *Chironexfleckeri* Southcott and *Carybdea* *marsupialis* from Puerto Rico [2,8,9,12,13]. Life-cycle studies on cubozoa indicate that unlike scyphozoa, cubozoa do not undergo strobilation; instead the entire polyp undergoes metamorphosis into a medusa [7,8,11,13–16], with exception noted in *Carybdea* *marsupialis* from Puerto Rico [17]. During the polyp stage, feeding and asexual reproduction occurs and continues until external factors, or cues, such as temperature [14,15,18–20] or light conditions [16], induce metamorphosis from a polyp to a medusa [1,2,21]. The medusae then grow to sexual maturity and may form predictable spawning aggregations [22].

It has been speculated that cubopolyps inhabit areas that do not allow for the annual persistence of the polyp, for example tropical estuaries which are commonly inundated through annual monsoonal rains [2,13]. The polyp may not be able to persist during this period due to extreme drops in salinity and temperature [2]. The monsoonal rains coincide with the seasonal abundance of at least one species of cubomedusae found in coastal areas [1]. It has also been speculated that the cubopolyps are forced to metamorphose to survive through the wet season in the medusa form, therefore capitalizing on two different environmental niches through two body forms seasonally [2,13]. Evidence of this process has been shown in coastal *Chiropsellabronzie* Gershwin where influxes of medusa correspond with recent rainfall events [1]. In Scyphozoa, many factors such as temperature [2,23–25], salinity [23–26], and food availability [12,13,25,27] have been shown to impact on asexual reproduction and survivorship of scyphistomae. Furthermore, factors such as temperature, salinity, food availability and light, have been shown to initiate strobilation [6,23,26,28–34],. As a result it is hypothesized that A. nr *mordens* polyps use thermal and osmotic cues in order to time, or trigger, the onset of metamorphosis. However, the factors that cause or contribute to the seasonality of cubozoa are currently unknown, and as polyps contribute to population numbers through asexual reproduction it has been suggested that the seasonal timing initiating metamorphosis plays a major role in the seasonality of cubozoan medusae [1,2].

A tropical oceanic species of cubozoa medusae*, A latina* nr *mordens*, can be reliably found in Australia’s Coral Seawaters in predictable spawning aggregations occurring eight to ten days after a full moon (Carrett & Seymour, unpublished data). In order to gain insight into the periodicity of A. nr *mordens* medusa, research into the ecology and physiology of the polyp stage and their responses to environmental factors, is required.

Temperature, salinity, and food availability are explored as contributors to fecundity and mortality of A. nr *mordens* polyps with aims to determine the thermal and osmotic parameters for A. nr *mordens* polyps. Additionally, this study aims to determine the influence of temperature, salinity, and food on asexual reproduction and metamorphosis of A. nr *mordens* polyps.

## Methods

### Species Description

The polyp culture used in this study was derived from sexually mature medusae of A. nr *mordens* collected from spawning aggregations at Osprey Reef (approximately 13º 54.190S 146º 38.985E) in 2005 and kept in culture for approximately five years. No specific permissions were required for these locations/activities, as the species involved is not endangered or protected and the collection area (Coral Sea) did not require permits to collect these animals. Individual medusae had bell heights of approximately 100 mm with tentacles up to 750 mm long, six eyes per rhopalial club, and kidney-shaped statoliths. This is the same location from which the type specimens of *A latina mordens* were collected [35]. However, the collected specimens do not share all the characteristics of the type description and are therefore referred to here as *A latina* nr *mordens* to indicate they are similar, but not identical to, *A latina mordens*.

### Experimental Design

This research was comprised of two separate experiments, which were each monitored for polyp survival and metamorphosis. The first experiment consisted of 64 thermal and osmotic treatments (see Method: Thermal and Osmotic Effects on Polyp Survival). Each treatment consisted of six independent replicates and each replicate consisted of a starting population of 50 polyps (i.e., sixty-four, six-well microplates, or 364 wells total, with 50 polyps housed in each well) (see Procedure). Polyps were only exposed to one treatment, there was no mixing of water between replicates or treatments, and all treatments were conducted simultaneously. These polyps were then observed for survival and metamorphosis.

A further four, six-well microplates were required to test the effects of feeding frequency on polyp survival and metamorphosis. There were six independent replicates of each treatment (i.e., four, six-well microplates, or 24 wells in total, with 50 polyps housed in each well) (see Method: Effects of Feeding Frequency on Polyp Fecundity and Mortality). Polyps were only exposed to one treatment, there was no mixing of water between replicates or treatments, treatments were conducted simultaneously, and were subsequently observed for survival and metamorphosis.

### Procedure

Prior to all experiments, 50 polyps were transferred from the culture to each well (approximately 11.5ml in volume) of six-well microplates containing filtered, artificial seawater, maintained at room temperature (24-26°C) and a salinity of 32.6‰. Polyps were allowed to acclimate in the microplates for approximately six weeks. During this time they were fed *Artemia* sp. nauplii once a week (approximately 50 *Artemia* per well). A complete water exchange using filtered artificial seawater took place 24 hours after each feeding event. During the acclimation period and experimental trials, a 13:11 light/dark cycle was maintained. Furthermore, during both of the following experiments, each feeding event also consisted of approximately 50 *Artemia* per well.

To allow comparisons between treatments the starting population of polyps in each replicate was recorded as 100% with subsequent measurements converted to a percentage of this starting measure. These data were arcsine transformed prior to analysis to normalise their distribution.

### Thermal and Osmotic Effects on Polyp Survival

To determine the thermal and osmotic tolerances of A. nr *mordens* polyps, and the effects these treatments had on survival, replicates of polyps (as described above) were exposed to a 64 combination matrix of eight temperatures (11, 14, 18, 21, 25, 28, 31, and 34ºC) and eight salinities (22.6, 25.9, 29.3, 32.6, 36.0, 39.3, 42.6, and 46.0‰) for a period of six weeks. The range of temperatures selected encompass the potential thermal regime a polyp may experience at Osprey Reef, from 34°C in shallow exposed coral hollows, to 11°C at depths of approximately 500 meters. Temperatures below this occur at Osprey Reef at great depth (water depth reaches 1000 meters), however, were not tested due to equipment restrictions. The salinity range tested includes the salinities common in the Coral Sea between 31‰ during heavy rain periods and 34‰ to 35‰ during normal conditions. In order to deduce the osmotic tolerances of A. nr *mordens* polyps, this range was expanded to include hypersaline and hyposaline conditions. Prior to the beginning of the experiments each replicate was photographed and the number of polyps in each well was determined. During the six week experiment, each replicate was photographed and the polyps were fed once per week.

To determine the limits of survivorship of polyps, mortality at each of the 64 combinations of temperature and salinity were graphed on a contour chart, with isopleths showing average percentage survivorship after six weeks exposure to the experimental conditions.

### The Effects of Feeding Frequency on Polyp Fecundity (Lateral Budding) and Mortality

In order to determine the effect of feeding frequency on polyp fecundity, polyps were exposed to a variety of feeding regimes at a constant temperature and salinity condition of 25ºC and 32.6‰, which was the treatment that yielded the highest fecundity. The feeding treatments consisted of feeding once every 3, 7, or 14 days, over a 27 day period. The replicates were photographed and the changes in population numbers (fecundity) were determined in three day intervals. An ANCOVA was performed to determine if there was a significant relationship between time and fecundity for the five feeding treatments (the covariate).

### Metamorphosis

To determine what factors may initiate metamorphosis for A. nr *mordens* from polyps to medusae, polyps were maintained at culture conditions of 27ºC and 33‰ and transferred into one of the 64 temperature and salinity combinations described. These polyps were monitored for signs of metamorphosis (i.e., change in body shape and colour, visible formation of statoliths, attached and free swimming medusae) weekly during the six-week experiment. At the end of this period, replicates were returned to near culture conditions of 25°C, 32.6‰ for four weeks. Weekly feeding, water replacement, and photographing continued while evidence of metamorphosis was recorded.

In order to determine if feeding frequency initiates metamorphosis, *A*. nr *mordens* polyps were exposed to four feeding treatments (fed every 3, 7, 14 day, and no food treatment) maintained at a constant condition of 25°C and 32.6‰. These treatments were monitored for evidence of metamorphosis, in three day intervals, over a 27-day period.

The number of polyps undergoing metamorphosis was then converted to a proportion of the starting replicate number. Data were analysis via two-way repeated measure ANOVA’s: one to test if salinity and/or temperature significantly affected metamorphosis, and the second to determine the effect that feeding and time had on the proportion of polyps undergoing metamorphosis. Where Mauchly’s test of sphericity was violated, Greenhouse-Geisser was reported. Post-hoc LSD, with Bonferroni adjustment for multiple comparisons was conducted on any significant treatment effects. All analysis was conducted using IBM SPSS Version 20.

## Results

### Thermal and Osmotic Effects on Polyp Fecundity (Lateral Budding) and Mortality

**Figure 1:**
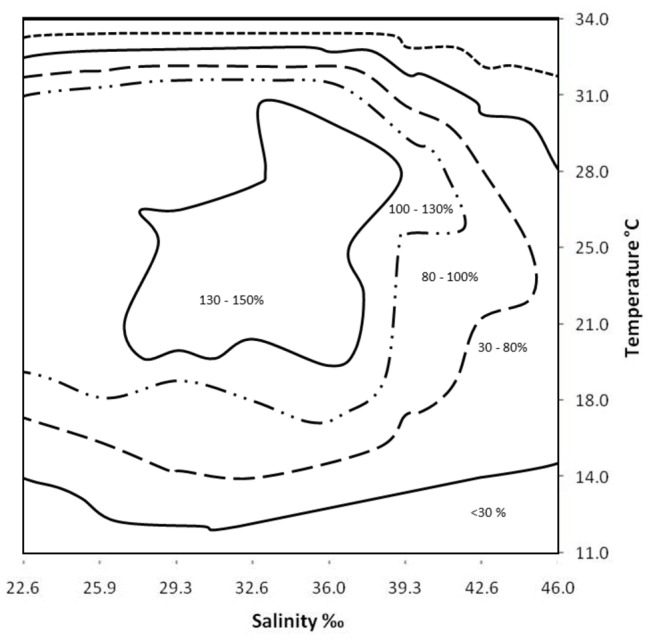
Mean polyp survivorship after six weeks exposure to a matrix of eight temperatures and eight salinities (64 treatments). Isopleths are defined as the percentage of the starting population present after six weeks, where values above 100% indicate population increase and values below 100% indicate population decrease.

The proportion of polyps present after six weeks exposure to the 64 treatments were significantly affected by temperature (*F*_7,320_ = 482.93, *p* < 0.001), salinity (*F*_7,320_ = 93.81, *p* < 0.001), and their interaction (*F*_49,320_ = 12.45, *p* < 0.001) (Figure 1). High temperatures (34°C) were associated with 100% mortality at all salinity treatments. Polyp survival was always positive between 18–31°C, in salinity ranges of 22 to 40‰. At temperatures from 11 to 14°C, at all salinity treatments, polyp survival rate was negative and ranged from 30 to 80%.

### The Effects of Feeding Frequency on Polyp Fecundity (Lateral Budding) and Mortality

**Figure 2:**
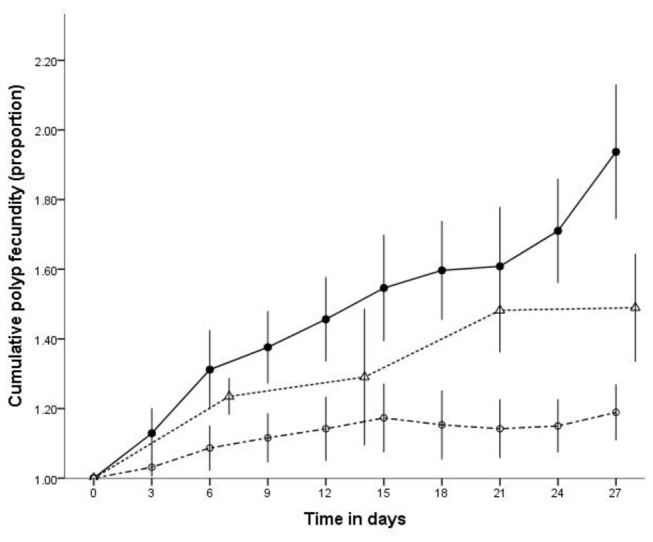
Mean cumulative polyp fecundity, in proportion, at three feeding levels over the 27 day experiment. The feeding levels consisted of: fed every 3 days ●; every 7 days Δ; every 14 days ○. Values are reported as means and error bars represent 95% CI.

There was a statistically significant difference (Figure 2) in the proportional population increase of polyps between the feeding treatments (*F*_1,256_ = 87.001, *p* < 0.001), and a significant difference over time (*F*_12,256_ = 13.341, *p* < 0.001). Polyps fed more regularly budded more often and produced more polyps with time.

### Metamorphosis

**Figure 3:**
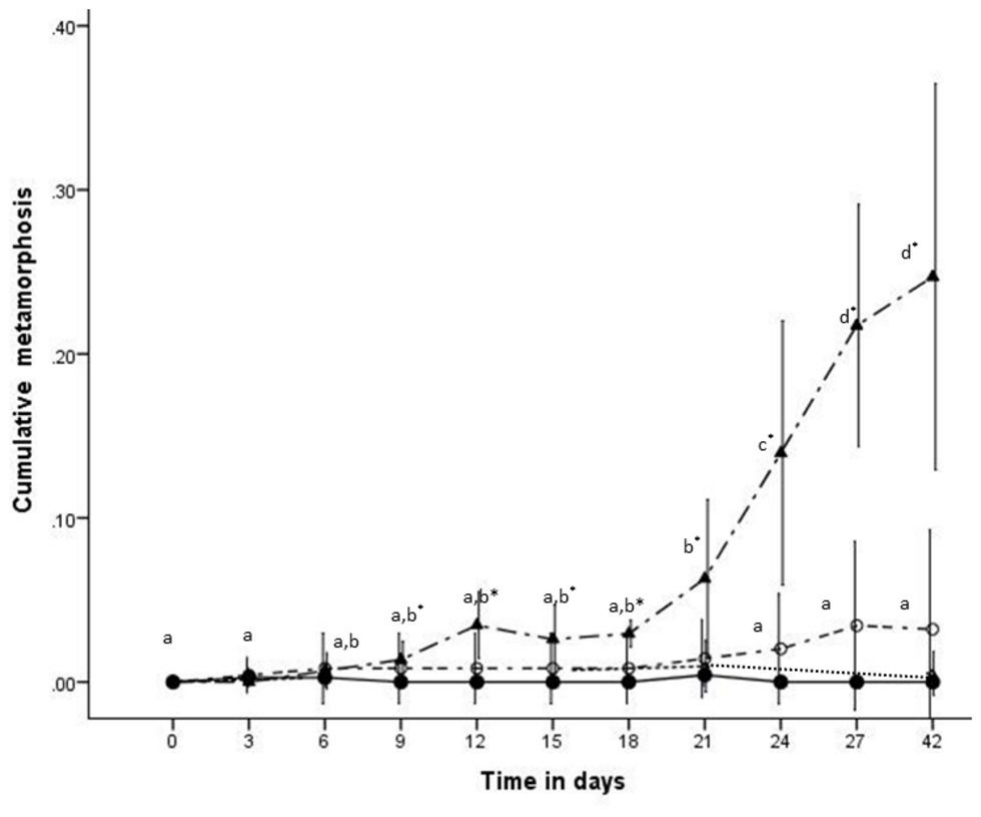
The proportion of polyps undegoing metamorphosis over time in each of the four feeding treatments. fed every 3 days ●; every 7 days Δ; every 14 days ○; no food **▲**. Values are reported as means and error bars represent 95% CI. Corresponding letters represent non-significant differences at alpha 0.05, and (*) indicates where metamorphosis is significantly different from zero.

Temperature, salinity, and all variations thereof, did not result in any metamorphosis, even when the conditions were returned to 25ºC and 32.6‰. However, there was a significant interaction of feeding frequency and time (*F*_16,80_ = 24.43, *p* < 0.001) (Figure 3) on polyp metamorphosis. A higher proportion of polyps underwent metamorphosis when not fed, 22% at the 27 day period, compared to all other feeding treatments, which were not significantly different from zero (*t*_23_ = 1.72, *p* = 0.099). In the no food treatment, metamorphosis became significantly different from zero on day nine (*t*_5_ = 3.13, *p* = 0.026).

## Discussion

*A latina* nr *mordens* polyps exhibited a population increase in temperature treatments between 18 and 31ºC. This range of temperatures occurs in depths between zero and 180 meters at Osprey Reef (Dunstan, 2009, unpublished data), below this depth it theoretically appears that the temperature is too low for population stability. Within this temperature range, the maximum population increase of A. nr *mordens* polyps occurred in thermal treatments between 21 and 25ºC, suggesting that this species should be found at depths of less than 120 meters on Osprey Reef, where these temperatures occur. Furthermore, the polyps also exhibited population increases at salinities between 22.6 to 39.3‰, which encompasses all expected salinity levels found in the Coral Sea (30 to 35‰) [36].

In addition to suitable thermal and osmotic conditions, polyps require hard substrate for attachment. *A latina* nr *mordens* are expected to be found in aggregations attached to the underside of hard substrate. This theory is based on research on other cnidarian polyps that revealed the preferences for the underside of rocks in order to avoid strong currents, siltation, and to reduce competition with other cnidarians and algae [2,37–40]. It is expected that competition for hard substrate on the reef will be much higher than competition for substrate in costal environments due to increased species richness and reduced available substrate.

Further exacerbating the competition for hard substrate is the process of asexual reproduction where feeding frequency has a positive effect on fecundity. In the high feeding frequency treatment there was an approximate 90% population increase over 27 days. We believe this process is continuous, leading to the potential for rapid population increases in polyp numbers.

Due to the periodicity of the medusae, it was expected that environmental cues would initiate metamorphosis; however, metamorphosis in A. nr *mordens* was not initiated by thermal or osmotic variations. In previous research on Scyphozoa, variations in temperature and/or salinity have been considered a primary cue for strobilation [2,6,23,24,41,42]. There is also evidence of an additive effect of environmental factors where variations in temperature, increased nutrients, and high sunlight, in combination, trigger strobilation [5,6,23]. This process has been shown to drive the seasonality of medusae in many species of Scyphozoa [5]. However, we believe, based on the data presented here, that A. nr *morden* polyps use a different strategy as a cue to initiate metamorphosis.

Continuous asexual polyp reproduction, coupled with restricted dispersal distances (tens of millimetres observed in *C* *. fleckeri* polyps) [2,13], and limited hard substrate options, would presumably lead to increased polyp density and may reduce food availability per polyp due to intraspecific and/or interspecific competition. Reduced food was found to induce metamorphosis in A. nr *mordens* polyps to medusae. This suggests that metamorphosis may be a survival strategy to avoid the deleterious effects of increased density and starvation where the medusae are able to source food from a different environmental niche. As such, *A*. nr *mordens* polyps maybe risk-spreading (i.e., bet-hedging), whereby the variation may be attributed to the associated disadvantages, or risks, involved in undergoing metamorphosis at a given time (e.g., the arrest of asexual reproduction reducing reproductive output, and the probability of reaching sexual maturity as medusae). Starvation may need to reach a critical level for an individual polyp before metamorphosis is initiated, which may explain why metamorphosis increased with time over the 27-day experiment. However, it is possible that there is an interaction between population density of polyps, environmental conditions, and food availability that determines metamorphosis and this should be investigated in future studies. This is the first study to investigate the environmental factors effecting cubozoa polyps and suggests that the factors that initiate metamorphosis of these polyps is distinctly different from that seen in strobilation of scyphozoans.
