# Correction: Seasonality in Polyps of a Tropical Cubozoan: *A latina* nr *mordens*

**DOI:** 10.1371/journal.pone.0151887

**Published:** 2016-03-14

**Authors:** The PLOS ONE Staff

Due to an error introduced during the typesetting process, the species name *A latina* nr *mordens* appears incorrectly throughout the article and in the article title. The correct species name should be *Alatina* nr *mordens*. The correct citation is: Courtney R, Seymour J (2013) Seasonality in Polyps of a Tropical Cubozoan: *Alatina* nr *mordens*. PLoS ONE 8(7): e69369. doi:10.1371/journal.pone.0069369. The publisher apologizes for this error.
